# Brain tumor classification from MRI scans: a framework of hybrid deep learning model with Bayesian optimization and quantum theory-based marine predator algorithm

**DOI:** 10.3389/fonc.2024.1335740

**Published:** 2024-02-08

**Authors:** Muhammad Sami Ullah, Muhammad Attique Khan, Anum Masood, Olfa Mzoughi, Oumaima Saidani, Nazik Alturki

**Affiliations:** ^1^ Department of Computer Science, HITEC University, Taxila, Pakistan; ^2^ Department of Physics, Norwegian University of Science and Technology, Trondheim, Norway; ^3^ Department of Computer Science, College of Computer Engineering and Sciences, Prince Sattam bin Abdulaziz University, Al−Kharj, Saudi Arabia; ^4^ Department of Information Systems, College of Computer and Information Sciences, Princess Nourah Bint Abdulrahman University, Riyadh, Saudi Arabia

**Keywords:** brain tumor, MRI, contrast enhancement, deep learning, hyperparameters optimization, feature selection

## Abstract

Brain tumor classification is one of the most difficult tasks for clinical diagnosis and treatment in medical image analysis. Any errors that occur throughout the brain tumor diagnosis process may result in a shorter human life span. Nevertheless, most currently used techniques ignore certain features that have particular significance and relevance to the classification problem in favor of extracting and choosing deep significance features. One important area of research is the deep learning-based categorization of brain tumors using brain magnetic resonance imaging (MRI). This paper proposes an automated deep learning model and an optimal information fusion framework for classifying brain tumor from MRI images. The dataset used in this work was imbalanced, a key challenge for training selected networks. This imbalance in the training dataset impacts the performance of deep learning models because it causes the classifier performance to become biased in favor of the majority class. We designed a sparse autoencoder network to generate new images that resolve the problem of imbalance. After that, two pretrained neural networks were modified and the hyperparameters were initialized using Bayesian optimization, which was later utilized for the training process. After that, deep features were extracted from the global average pooling layer. The extracted features contain few irrelevant information; therefore, we proposed an improved Quantum Theory-based Marine Predator Optimization algorithm (QTbMPA). The proposed QTbMPA selects both networks’ best features and finally fuses using a serial-based approach. The fused feature set is passed to neural network classifiers for the final classification. The proposed framework tested on an augmented Figshare dataset and an improved accuracy of 99.80%, a sensitivity rate of 99.83%, a false negative rate of 17%, and a precision rate of 99.83% is obtained. Comparison and ablation study show the improvement in the accuracy of this work.

## 1 Introduction

One of the deadliest brain disorders is a brain tumor, which develops from an abnormal development of tissue inside the skull. Primary and secondary forms can be distinguished among them. 70% of cases of primary brain tumors only spread within the brain (1). In contrast, secondary tumors start in an organ like the breast, kidney, or lung before metastasizing to the brain (2). The World Health Organization (WHO) divides malignant gliomas into two categories: grade IV/IV tumors, which include glioblastoma multiforme (GBM), and grade III/IV tumors, which include anaplastic astrocytoma, anaplastic oligodendroglioma, anaplastic oligoastrocytoma, and anaplastic ependymomas. With an incidence rate of 3.19 cases per 100,000 person a year and a median age of 64, GBM is the most prevalent malignant brain tumor. It makes up 80% of all primary malignant CNS tumors and 45.2% of all malignant CNS tumors. GBM is 1.5 times more common in men than in women, and it is twice as common in white people as it is in black people (3).

Meningioma is the most common primary tumor of the central nervous system, with 5/100,000 annual occurrence. Radiation therapy and hormone use are risk factors. According to the WHO’s 2016 histological criteria, the majority of meningiomas are grade I benign tumors; however, up to 15% can be atypical and 2% can be anaplastic (4). Pituitary adenomas usually are benign tumors that develop from unusual pituitary gland cell development. They appear either by producing too much hormone or by putting pressure on the surrounding structures, which causes less hormone to be secreted. Prolactinomas, non-functioning adenomas, adenomas that secrete growth hormone, and adenomas that secrete adrenocorticotrophic hormones are the four primary forms. Less frequent kinds include gonadotroph adenomas with clinically significant luteinizing hormone, follicle-stimulating hormone secretion, and thyroid-stimulating hormone-secreting adenomas. Pituitary incidentalomas are a subtype that was unintentionally found while undergoing brain MRI. They can be divided into macroadenomas (bigger, accounting for roughly 40% of occurrences) and microadenomas (less than 1 cm in diameter). Macroadenomas can strain essential structures and regions like the optic chiasm (5).

Gliomas and meningioma emerge from neuroglial and brain membranes, respectively; both are the most frequent primary brain cancers. Pituitary gland and nerve sheath tumors are also included in this group. High-grade gliomas are a common form of malignant tumor. Meningiomas are typically benign; however, they can occasionally turn cancerous (6). Gliomas are more common in men, whereas meningiomas are more common in women; other brain cancers affect both sexes equally (7). Pituitary tumors, whether benign or malignant, can have severe consequences due to their location. Malignant tumors spread quickly, whereas benign tumors develop slowly and are generally entirely eradicated through surgery (8).

**Figure 1:**
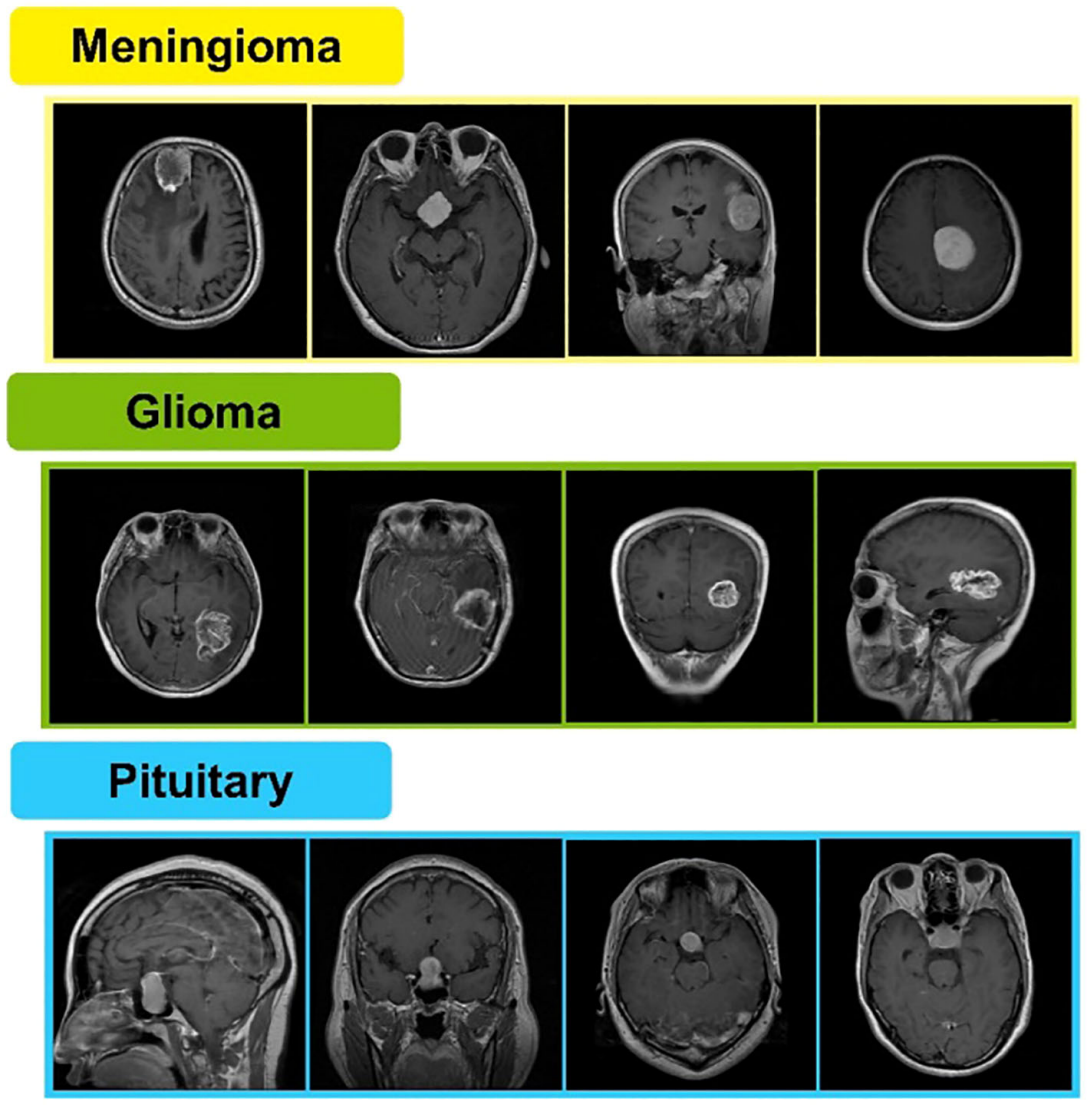
Sample MRI images of brain Meningioma, glioma, and pituitary tumors.

Radiologists and clinicians have substantial difficulties in detecting brain tumors. Brain tumor images produced in medical settings might be challenging to analyze. As a result, there is a need for computer-aided procedures with increased early detection accuracy. Currently, there is a lot of interest in using multimodal images to classify brain tumors (9). Magnetic resonance imaging (MRI) is frequently used to diagnose brain malignancies. A tumor can be found via MRI, commonly used to identify brain tissues based on their size, shape, or location (10). Figshare is a publicly available MRI image-based brain tumor dataset containing 3,064 T1-weighted contrast-enhanced images. These are obtained from 233 patients. A total of 1,426 slices of glioma, 708 slices of meningioma, and 930 slices of pituitary tumors are included in said dataset (11, 12). A few sample images are shown in **Figure 1**.

In recent years, interest in computer vision has grown across various fields of studies, from medical to industrial robotics. Computer science and advances in image processing techniques have greatly aided computer vision (13). Deep learning is a diverse set of techniques that includes neural networks, hierarchical probabilistic models, and a wide range of unsupervised and supervised feature learning algorithms. Deep learning approaches have recently gained popularity because of their ability to beat prior state-of-the-art techniques in various tasks and the amount of complex data from various sources (e.g., visual, auditory, medical, social, and sensor) (14). Deep learning has made significant advances in a wide range of computer vision tasks, including object recognition (15), motion tracking (16), and medical image classification and detection (17, 18). Classification of brain tumors for medical specialists is an important field where computer vision and deep learning techniques work together and bring prosperity to patients with non-invasive diagnosis of brain tumors using MRI.

### 1.1 Aims and objectives

Image acquisition from MRI has loss of information that leads to improper feature visibility. A technique is required to employ that can enhance the contrast of MRI images so that loss of information during the acquisition process can be minimized. Hence, feature visibility can be improved and classification problems can be addressed, which has a close relationship with feature visibility. In order to address classification problems for MRI images of brain tumors, there is an immense need to introduce a technique using state-of-the-art deep learning methods. In a quest to fulfill this need, a deep learning technique should acquire brain tumor MRI images from publically available benchmark datasets. The selected dataset explained in a related section of this manuscript has significant imbalance classes, so it is important to incorporate a data augmentation technique that can gracefully fill the gap of imbalanced dataset classes. After enhancement of contrast and data augmentation steps, lightweight pretrained deep leaning models need to be deployed and modified based on the low complexity for training of the balanced dataset. Optimization of hyperparameters to train deep learning models is required to select the optimal combination of values for model training on the selected dataset. Extracted features can be optimized using some optimization algorithms and then be fused together. Feature fusion greatly impacts the overall classification accuracy. The subsequent section presents the major challenges in order to develop an aimed technique and contribution to address these challenges in proposed work.

### 1.2 Major challenges and contributions

This imbalance in the training dataset impacts the performance of deep learning models because it causes the classifier performance to become biased in favor of the majority class. The authors tried to resolve this issue by using few traditional techniques such as flip image and rotate image, and few of the authors performed contrast enhancement. However, these techniques are not enough, and the images are highly duplicated. Therefore, it is essential to address this challenge by employing some of the latest techniques, such as GAN and encoders. Still, most currently used feature selection techniques ignore certain features that have particular significance and relevance to the classification problem in favor of extracting and choosing deep significance features. We proposed a hybrid deep learning framework with BO and QTbMPA feature selection algorithms to address these challenges. Our major contributions are listed below.

▪ Sparse Autoencoder architecture was proposed for the generation of new images based on the training data for the augmentation process.
▪ Two lightweight pretrained deep learning models were fine-tuned based on the additional layers and removal of pooling layers. The models were trained from scratch on an augmented dataset.
▪ A Bayesian optimization technique was implemented to initialize the hyperparameters of the fine-tuned deep models for improved learning.
▪ An efficient Quantum Theory-based Marine Predator Optimization algorithm was proposed for the selection of best features for the final classification.
▪ A detailed ablation study was performed on the proposed framework for the validation of the proposed framework.

## 2 Literature review

A wide range of classification approaches have been introduced for the Figshare dataset. Several techniques have been introduced in the literature for the classification of brain tumor from MRI images. Researchers used deep learning models for the feature extraction and later performed classification using Softmax and machine learning classifiers. A novel deep transfer learning-based model was identified by Alanazi et al. (19). It entails creating several convolutional neural network models and then utilizing transfer learning to repurpose a 22-layer model for subclass classification. The proposed model achieved 95.75% accuracy on three classes of the Figshare dataset. Moreover, the technique was also validated for an unseen dataset and achieved an accuracy of 96.89%. Another DeepTumorNet hybrid deep learning model was suggested by Raza et al. (20). The last five layers of GoogleNet were eliminated while creating the hybrid DeepTumorNet technique, and 15 new layers were added. They used the feature map’s leaky ReLU activation function to make the model more expressive. The suggested model was evaluated on the Figshare dataset and achieved 99.67% accuracy. Tummala et al. (21) used ensemble-oriented vision transformer-based pretrained models to classify the modalities of the Figshare dataset. An ensemble of B/16, B/32, L/16, and L/32 was used. The selected approach achieved an overall accuracy of 98.70%. Attention mechanism, patch-oriented input, and token embedding are techniques used in vision transformers, which make them more computationally expensive, and processing requires a tensor processing unit (TPU) environment.

Another work by Polat et al. (22) introduced a novel divergence-based feature extractor which is used for classification by decreasing weights for deep neural networks. The achieved accuracy was 99.18%. They have reduced the input image dimensions considerably (i.e., 512× 512 to 128 × 128) , which can result in loss of spatial information. Loss of information at the input level can result in compromised accuracy. A technique that uses a multilevel attention network (MANet) (23) was suggested by Shaik et al. in which the model has an attention mechanism with several tiers of attention blocks and can concentrate on crucial spatial and category-specific properties. Prioritizing tumor details in the image is done by the first attention block, and the second attention layer is highlighted by the tumor-specific descriptors using ConvLSTM. MRI images are represented as input to the model using pretrained features from the XCeption network. The resultant accuracy of 96.51 for the Figshare dataset was obtained. In the presented technique, only those glioma images with tumor in it will be classified. A CNN-based approach was created by Haq et al. (24); they performed classification as well as segmentation. A classification accuracy of 98% was achieved. The proposed algorithm has a long running time and needs an improvement to reduce the running time. In another technique, Rahman et al. (25) implemented a Parallel Deep Convolutional Neural Network (PDCNN) technique. It operates in two concurrent stages to capture both global and local features. The model includes dropout regularization and batch normalization to alleviate the overfitting issue. The classification accuracy is 97.60%. The proportion of 80:20 training and testing data was respectively used. A major proportion of training data may lead to overfitting as it becomes specialized for known data but not for unseen or unknown data.

The authors Talukder et al. (22) presented a technique to classify brain tumors. They used different pretrained models and obtained an accuracy of 99.68% on ResNet50V2. The lack of sharp images is the main shortcoming of this study. In their work, Aloraini et al. (26) presented another technique in which the authors utilized a hybrid method combining a transformer with an attention mechanism to capture global features. Local features were extracted using a convolutional neural network (CNN). The approach attained an accuracy of 99.10% for the Figshare dataset. Few misclassifications were reported due to visual similarity between classes. In their work, authors Athisayamani et al. (27) introduced a new adaptive Canny Mayfly algorithm for edge identification. An algorithm that reduces the dimension of retrieved features, the enhanced chimpanzee optimization algorithm (EChOA), is utilized to choose features. The feature classification process is then done using the Softmax classifier and ResNet-152. The proposed technique achieved an accuracy of 98.85%. In their presented work, the authors Mishra et al. (28) provided a method for classifying brain tumors using a K-NN classifier, where the parameter "k" is adjusted and the best feature set is selected using the binary version of the comprehensive learning elephant herding optimization (CLEHO) algorithm. The presented method obtained an accuracy of 98.97%, better than the recent techniques. A pretrained model-based approach was suggested by the authors Malla et al. (25), in which a transfer learning DCNN framework known as VGGNet was used. They employed transfer learning aspects such as fine-tuning the convolutional network and freezing layers for better performance. Features were extracted from the Global Average Pooling (GAP) layer. The technique resulted in an accuracy of 98.93% on the Figshare dataset. In the given approach, the feature dimensionality issue was not addressed, and that intended to address it in future research.

In another work, authors Cinar et al. (29) presented a Convolutional Neural Network (CNN) architecture for brain tumor classification. The model was compared with ResNet50, VGG19, DensetNet121, and InceptionV3 pretrained models. The presented model achieved an average classification accuracy of 98.32% on the prescribed dataset. The authors determined to enhance their technique using area and size-oriented metrics. In another technique, the authors Deepak et al. (30) coined an approach in which they trained CNN using three different methods: cross-entropy loss, class-weighted loss, and weighted local loss. They fused the features, and classification was performed with an accuracy of 95.40%. Another approach by authors by authors Zulfiqar et al. (31) suggested an approach in which five variations of the EfficientNets family’s pretrained models, EfficientNetB0 through EfficientNetB4, were fine-tuned. They also investigated how data augmentation affects the model’s accuracy. The best model’s attention maps are finally visualized using Grad-CAM, successfully highlighting the tumorous region of the brain cell. The achieved accuracy was 98.86%.

## 3 Methodology

**Figure 2:**
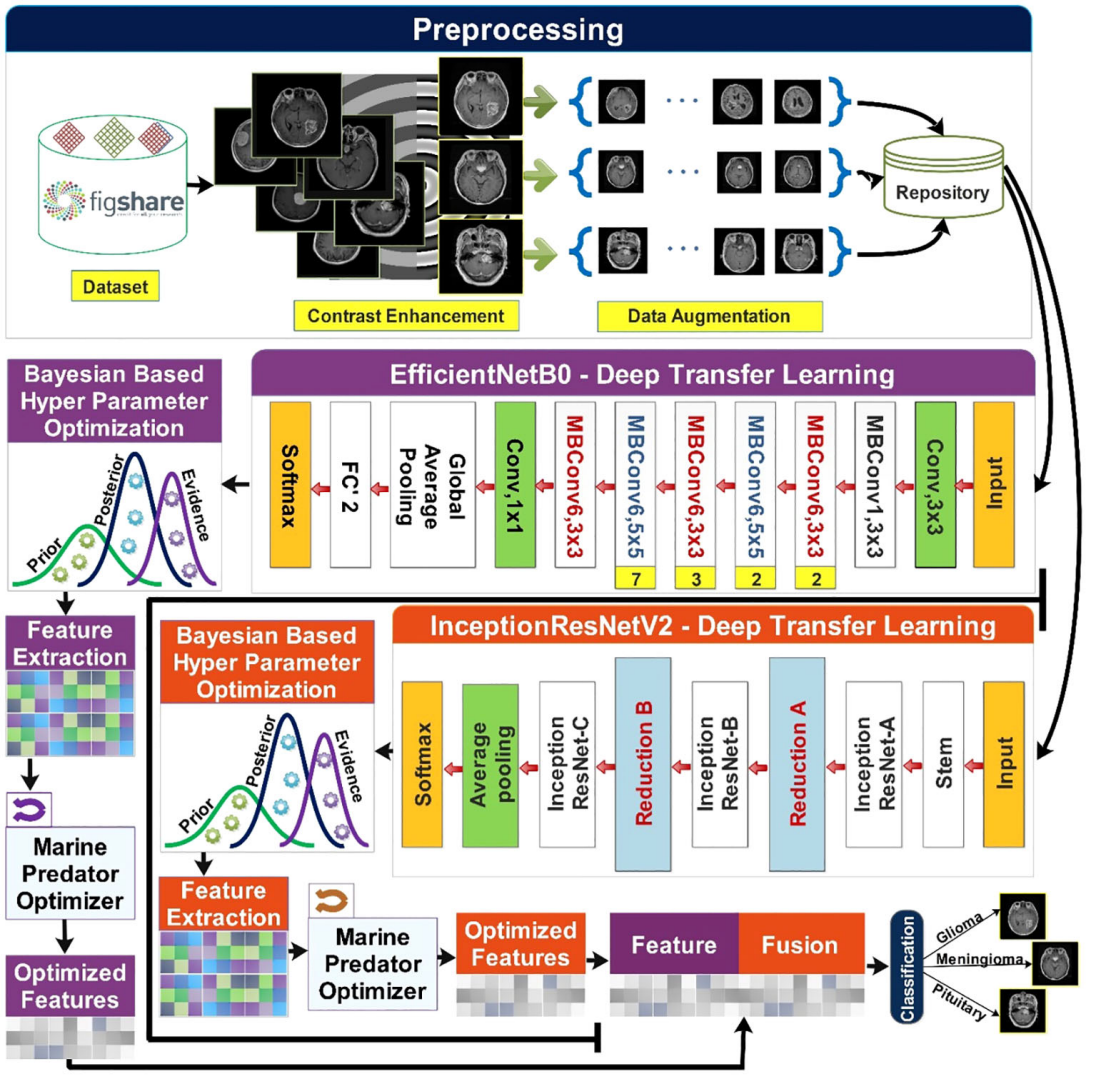
Proposed methodology of brain tumor classification using deep learning and optimization algorithm.

The proposed methodology of brain tumor classification is illustrated in **Figure 2**. This section starts with the preprocessing phase in which the Figshare brain tumor dataset (32) is obtained. The contrast enhancement step is crucial to improving the quality of low-contrast images, and it was performed using a statistical technique presented in (33). Data augmentation is performed on contrast-enhanced images. This step is taken into account due to the high imbalance of classes in the original dataset. Augmentation of the data is performed using sparse autoencoders (34). The said technique augments the data by learning the most important features of the original data and leaving behind the least important features. Two pretrained models named InceptionResNetV2 (35) and EfficientNetB0 (36) are used and fine-tuned for the input of preprocessed data. Dynamic and optimized selection of hyperparameters of both models is carried out using Bayesian-based optimization (37). Features are extracted from each optimized resultant model. To further optimize the features, a nature-inspired algorithm named the Marine Predators Algorithm (MPA) (38) is used on the obtained features of each model. Feature fusion is carried out, final classification is performed.

### 3.1 Dataset of this work

The Figshare dataset includes 3,064 T1 weighted contrast-enhanced MRI scans collected from 233 patients. There are three classes of these scans named meningioma, glioma, and pituitary, with 708, 1,426, and 930 MRI scans, respectively, in each class (32). Meningiomas are the most prevalent intracranial tumor, accounting for more than one-third of all primary central nervous system (CNS) tumors. They are typically benign tumors that can be observed or preferentially treated with extensive complete resection, which results in satisfactory outcomes. Meningioma with complex histology or in vulnerable areas has proven difficult to treat and predict prognostic outcomes (39).

Gliomas are divided into different categories based on the cells of their origin. They make up around 80% of all malignant primary brain tumors and are most frequent malignancies of the central nervous system (CNS). The most dangerous and common variety of glioma is called glioblastoma multiforme (GBM). More than 60% of adult brain tumors are caused by it. Despite the wide range of contemporary treatments available, GBM remains a fatal condition with a very bad prognosis. The median survival time for patients is typically 14 to 15 months after diagnosis of the deadly disease (40).

The anterior pituitary gland is the site of tumors called pituitary adenomas. They rank as the third most frequent adult cause of central nervous system malignancies (CNS). Most benign adenomas cause either a large-scale effect or an increase in hormone release. Depending on their size and hormone produced, pituitary adenomas appear differently in clinical evaluations (41). Samples of meningioma, glioma, and pituitary brain tumors from the Figshare dataset are presented in **Figure 1**.

### 3.2 Contrast enhancement

Analyzing medical images is challenging because of the inherent qualities present in medical images, such as poor contrast, speckle noise, signal dropouts, and complicated anatomical formation. Contrast enhancement is a vital component of subjective evaluation of image quality that aims to improve the overall excellence of medical imagery for feature visualization and clinical measurement (42). In fact, despite technological advancements in healthcare systems, they still produce images that demonstrate a deficiency in contrast due to improper locales and equipment limitations. To enhance the contrast of MRI images of the dataset discussed above, an existing technique for contrast enhancement (33) is employed. It uses basic statistics and some basic image processing methods. The approach adjusts the global and local contrast of a given image separately, then combines both results using logarithmic image processing (LIP), producing an output that is further analyzed by an adaptive linear stretching method to produce the improved version of the image. The overall process of contrast enhancement is defined as follows:

Letting a low-contrast image ${ɡ}_{\left(x,y\right)}$ , at first, the local contrast is altered using contrast stretching transformation (CST). The CST process is defined in Equation 1.


(1) $$k_{\left(x,y\right)}=\frac{1}{1+{\left(m/{ɡ}_{\left(x,y\right)}\right)}^{E}}$$

In the above equation, $k_{\left(x,y\right)}$ is the output of the CST procedure where x,y represents the dimensions of an image. The slope of the function is set by constant E , and its value is set to 0.5 for this experiment. The mean value of the input image is represented by m . A standard logic function is applied to the original image to change its global contrast. Mathematically, it is defined in Equation 2.


(2) $$j_{\left(x,y\right)}=\frac{exp\left({ɡ}_{\left(x,y\right)}\right)}{1+exp\left({ɡ}_{\left(x,y\right)}\right)}$$

The resultant images with altered local and global contrast will be combined. The Logarithmic Image Processing (LIP) method devised in (43) is for this purpose and is mathematically defined as follows:


(3) $$f_{\left(x,y\right)}=\frac{k_{\left(x,y\right)}+j_{\left(x,y\right)}}{1+\left(k_{\left(x,y\right)}*j_{\left(x,y\right)}\right)}$$

An exponent W is used to control the enhancement, and the entire equation is raised to its power of it. The scalar parameter (W>0) and its higher value lead to achieving a good level of contrast enhancement. Mathematically, it is defined in Equation 4.


(4) $$f_{\left(x,y\right)}={\left[\frac{k_{\left(x,y\right)}+j_{\left(x,y\right)}}{1+\left(k_{\left(x,y\right)}*j_{\left(x,y\right)}\right)}\right]}^{W}$$

Contrast enhancement of the image has been achieved after employing Equation 4, but the image $f_{\left(x,y\right)}\;$ does not correspond to the natural range of pixel values. A linear stretching method with adaptive form (40) brings a natural range of pixel values to the image. Mathematically, it is defined by Equation 5.


(5) $$t_{\left(x,y\right)}=\;\alpha *\;f_{\left(x,y\right)}-\beta$$

where $t_{\left(x,y\right)}$ is a resultant image, and α and β are the control variables for the stretching process. The value of these control variables is adjusted manually, but here, Equation 6 and Equation 7 are used to select the values of these variables automatically.


(6) $$\alpha =\frac{1}{max\left(f_{\left(x,y\right)}\right)\;-\;min\left(f_{\left(x,y\right)}\right)}$$

(7) $$\beta =\frac{min\left(f_{\left(x,y\right)}\right)}{max\left(f_{\left(x,y\right)}\right)\;-\;min\left(f_{\left(x,y\right)}\right)}$$

**Figure 3:**
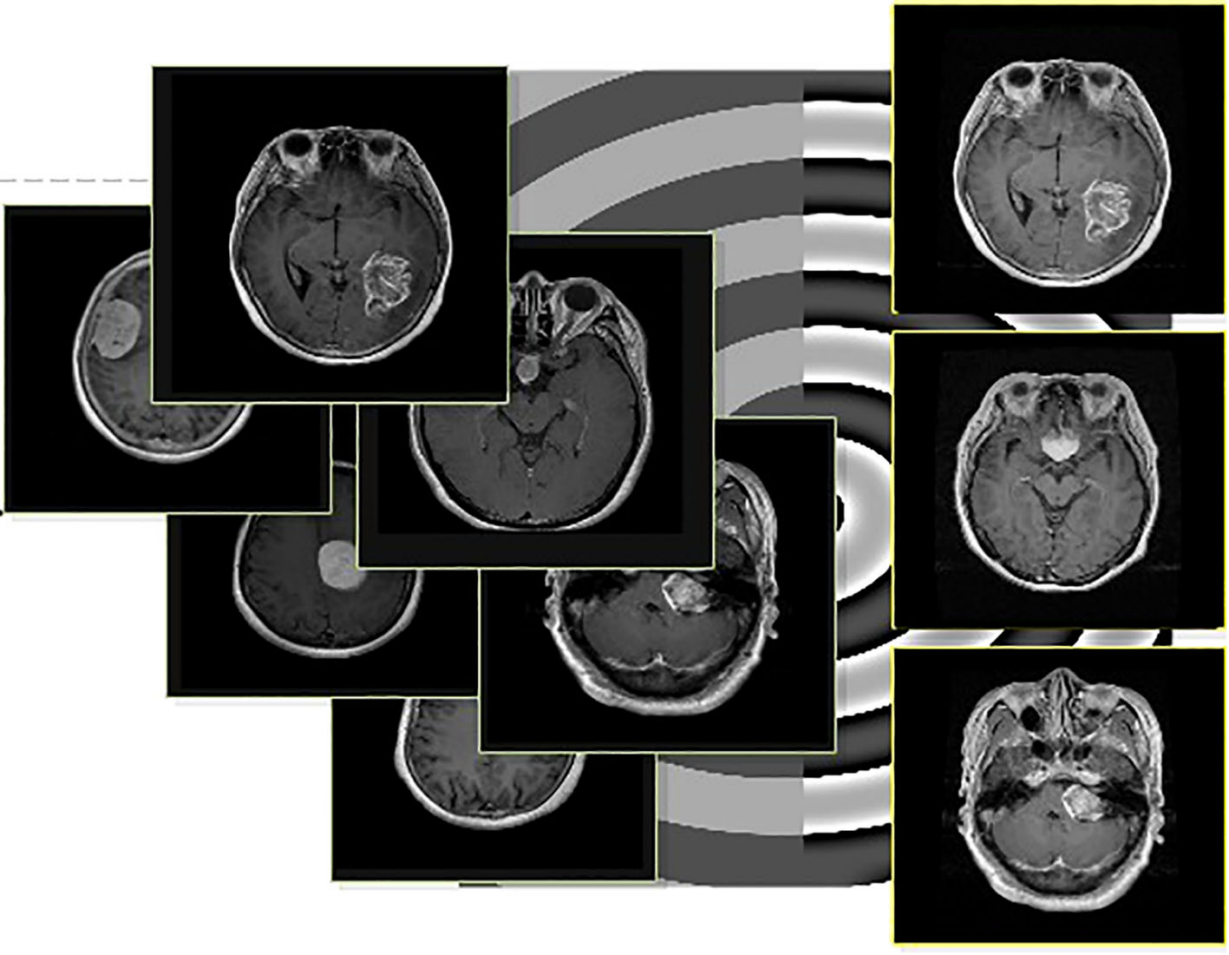
Visual illustration of the contrast enhancement process. The left images are original, and the right images are generated using contrast enhancement.

In the above equations, the variables max and min represent the upper and lower bounds of values for pixels of an image $f_{\left(x,y\right)},\;$ respectively. The pseudocode of the above mathematical description is given under **Pseudo-code 1.** A few visual images are also illustrated in **Figure 3**.

Pseudo Code 1 **Proposed Contrast Enhancement Technique.**

**Input:** Original image 
$g_{\left(x,y\right)}$
 and a parameter *W*.
Computation of CST method by using Equation 1.
Estimation of SL function by using Equation 2.
Calculation of modified LIP method by using Equation 4.
Computation of parameters *α* and *β* by using Equations 6 and 7

Processing of contrast by using Equation 5.
**Output:** Contrast Enhanced image 
$t_{\left(x,y\right)}$

### 3.3 Data augmentation

Classification performance is negatively impacted by class imbalance. The impact of imbalance on classification performance gets more robust with increasing task size. The effect of imbalance depends on the distribution of observations (i.e., images) throughout the classes and cannot be solely attributed to a lower overall number of training cases (44). In Section 3.1, it is noted that our dataset has a high-class imbalance. Hence, creating a dataset bias may lead to an overfitting problem for some classes. To fill that gap, we employed a sparse autoencoder (45) to augment the dataset instead of traditional methods.

Sparse Autoencoders learn a compressed representation of the input data. The following hyperparameters are used to train a sparse autoencoder network:

**Table:** 

Hyperparameters	Value
**Hidden size**	300
**Maximum epochs**	2000
**L2WeightRegularization**	0.001
**SparsityRegularization**	4
**SparsityProportion**	0.15

Hidden size parameter represents the number of neurons in layers. Few dozen neurons are enough for simpler tasks, but in order to use it with complex tasks, a few hundred neurons are used. A hidden size of 300 might be able to prevent overfitting while still having sufficient capacity to learn from the data, particularly in situations where bigger hidden sizes could cause overfitting.

One whole cycle through the whole training dataset is referred to as an epoch. The hyperparameter for maximum epochs indicates the maximum number of times the training dataset will be processed by the learning algorithm. In the proposed technique, the training dataset for augmentation took 2,000 epochs to converge at a suitable result for MRI images.

The intensity or weight of L2 regularization given to a neural network’s weights during training is commonly denoted by the hyperparameter L2WeightRegularization, which has a value of 0.001. The selection of 0.001 maintains a balance between letting the model learn from the data and regularizing it to avoid overfitting. It is also referred to as weight decay.

The sparsity regularization weight that is given to a neural network during training is indicated by the hyperparameter SparsityRegularization, and the chosen value for it is 4. By encouraging the model to have fewer active (non-zero) weights, the objective is to cause the weight matrices to become sparse, which means that during the training phase, a large number of the weights are driven to be zero or almost zero. Sparsity regularization helps to create a more effective and sparse representation for better feature selection.

The hyperparameter of “SparsityProportion” with a value of 0.15 commonly refers to a threshold sparsity level, which is used with sparsity regularization. The target of around 15% of the neural network’s weights becoming zero or almost zero is indicated by the value of 0.15. The sparsity regularization hyperparameter sets a threshold of 4, and weights that are below threshold are settled to zero. The value of 0.15 represents the proportion of weights that should actually fall below the specified threshold value during the training process.

The specified values for each hyperparameter are adjusted for augmentation of MRI images. The resultant images obtained from this step are used to augment the data.

**Figure 4:**
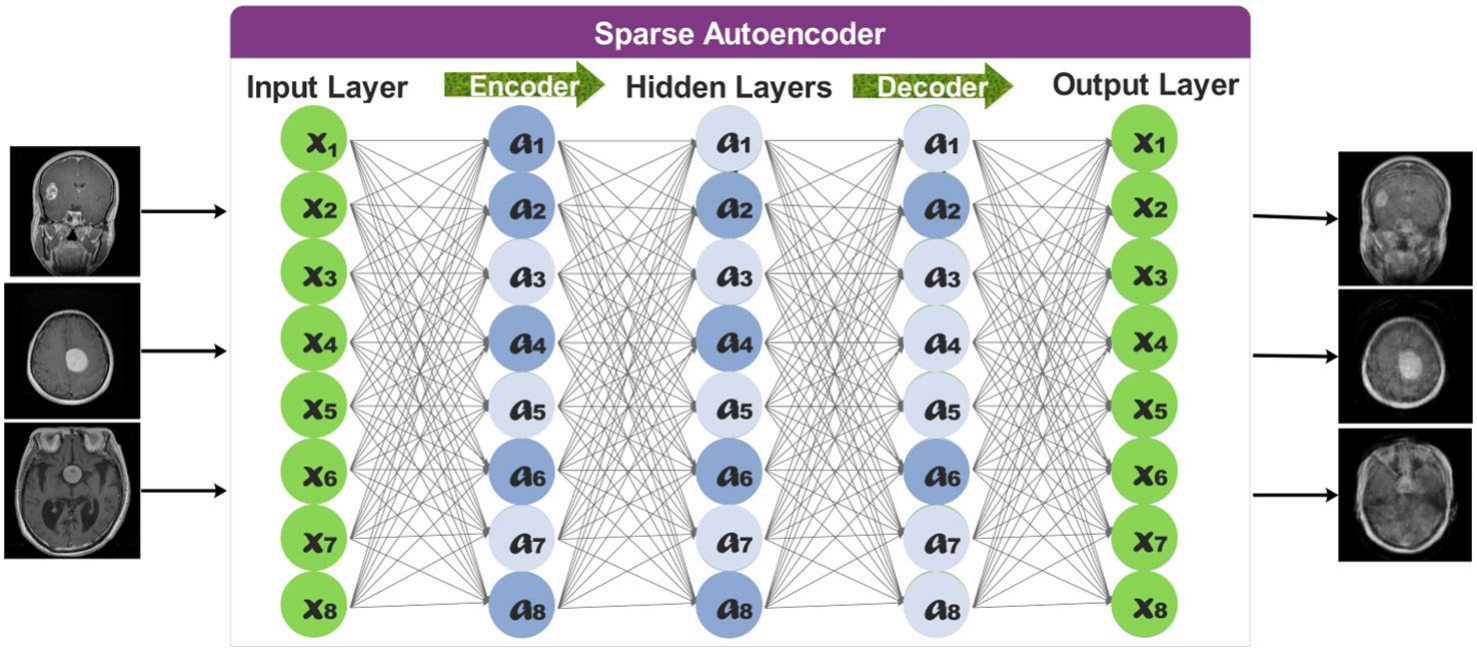
Representation of sparse autoencoder for data augmentation.

The overall representation of sparse autoencoders is provided in **Figure 4**.

The total number of observations for each class increased to 2,000 after employing the proposed sparse encoder network. The newly generated images have been utilized to train selected deep learning models.

### 3.4 Hyperparameter selection for modified EfficientNetB0 and InceptionResNetV2

The augmented dataset is used to train fine-tuned deep-learning models. Three hyperparameters for both models are optimized using Bayesian optimization to train the models. These hyperparameters are named InitialLearnRate, Momentum, and L2Regularization. The dynamic tuning of hyperparameters is a crucial task for deep learning models. In this case, dynamically selected values for specific hyperparameters are used until a specific best-value threshold is achieved. The particular model is then trained, and features are extracted for classification tasks.

Bayesian optimization (BO) is an effective technique for hyperparameter tuning. Implementation (46) can be achieved by setting an optimization goal. The Equations 8 and 9 below describes the BO process.


(8) $$x^{\prime }=arg\;\underset{xєA}{\max}f\left(x\right)$$

In the equation, search space is A for input x . BO is based on the Bayes theorem that is mathematically defined as follows:


(9) P(D│F)∝ P(F│D)P(D)

Given that an event or hypothesis F has occurred, it is the likelihood that the event or hypothesis D will also occur, where F denotes the evidence data, D denotes the model, and P(D│F) is the posterior probability that is proportional to the likelihood P(F│D) and is multiplied with a probability of D . The foundation of BO is the combination of sample data (evidence) and the prior distribution of the function f(x) to produce the posterior of the function. Then, based on the criterion, the posterior information is used to determine the location where the function f(x) is maximized. The criterion is also called an acquisition function (v) and is used to estimate the next sample point. Sampling points are searched using exploration and exploitation sampling methods while searching the sampling space. Exploration tends to search for sampling areas with high uncertainty. Exploitation searches for those samples that are of high value. These methods improve the performance, even with multiple local maxima solutions.

The prior distribution of the function f(x) , a crucial component in the statistical inference of the posterior distribution, is a requirement for Bayesian optimization in addition to sample information. The posterior distribution is updated using the Gaussian process to better align with the data, improving our forecasts’ accuracy and knowledge. **Algorithm 1** describes the working of BO.

Algorithm 1 **Bayesian optimization.**


1: For 
i=1,2,…

2: Find 
$x_{i}$
 by optimizing the acquisition function 
v
 over function 
f
: 
$x_{i}=arg\;\underset{x}{\max v}(x│D_{1:i-1})$
. **(Equation 10)**
3: Sample the objective function: 
$y_{i\;}=f\left(x_{i}\right)$
.
4: Augment the data 
$D_{1:i\;}=\;\left\{D_{1:i-1\;},\;\left(x_{i},y_{i}\right)\right\}$
 and update the posterior of function 
f
.
5: End For.

The algorithm consists of two parts: acquisition function maximization using step 2 and posterior distribution update using steps 3 and 4. Furthermore, the training dataset is denoted by $D_{1:i-1\;}={\left\{x_{n},y_{n}\right\}}_{n=1}^{i-1}\;$ with i−1 observations of function f . Each processed observation updates the posterior distribution. The updated distribution helps to find the highest value of the acquisition function at some point, which is then added to the training dataset. This process continues until the maximum number of iterations is reached or the difference between the current and best values so far is less than a predetermined threshold. The following starting and stopping criteria are selected for experiments. Number of seed points = 4; Maximum Objective Evaluation = 30, and Maximum time = Infinite by default.

A Gaussian process prior with additional Gaussian noise in the observations serves as the fundamental probabilistic model for the objective function f . Therefore, the Gaussian process with mean μ(x;θ) and covariance kernel function $k\left(x,x^{\prime },\theta \right)$ represent the prior distribution on f(x). Here, x represents the initial value, x' denotes the updated value, and θ is a parameter containing a kernel vector vector. Therefore, looking into more detail, we show a set of points x= x' with associated objective function $F=\;f_{i}$ and the prior joint probability distribution of the function value $k\left(x,x^{\prime }\right)$ where $k_{ij=}k\left(x_{i},x_{j}\right)$ and initially μ=0 . Moreover, Gaussian noise is added, which is denoted by $\sigma^{2}$ so the prior distribution has covariance $k\left(x,x^{\prime },\theta \right)+\sigma^{2}x,\;$ and therefore, the final Gaussian process regression is depicted by the following **Equation 11**.


(11) $$k\left(x_{i},x_{j},\theta \right)=\sigma_{f}^{2}\;exp\left[-\frac{1}{2}\;\sum_{m=1}^{d}\frac{{\left(x_{i},x_{j}\right)}^{2}}{\sigma_{m}^{2}}\right]$$

where $\sigma_{m}\;$ is length scale prediction m and m=1,2,3,…d , $\sigma_{f}$ is the signal standard deviation, $\theta_{m}=\log\left(\sigma_{m}\right),\;\theta_{d+1}=\text{log}\left(\sigma_{f}\right)$ , and $k\left(x,x^{\prime },\theta \right)$ is a Kernel function that significantly affects the quality of Gaussian process regression. In Bayesian optimization, the ARD Matern 5/2 kernel is optimized by default and is given in the following **Equation 12**.


(12) $$k\left(x_{i},x_{j}│\theta \right)=\sigma_{f}^{2}\left(1+\sqrt{5}\;r+\frac{5}{3}\;r^{2}\right)exp\left(-\sqrt{5}\;r\right)$$

where $r=\sum_{m=0}^{d}\sqrt{\frac{{\left(x_{im}-x_{jm}\right)}^{2}}{\sigma_{m}^{2}}}\;$ . BO employs the acquisition function to derive the highest value of the function f after collecting the posterior distribution of the objective function. Typically, we believe the large value of the objective function f matches the high value of the acquisition function. Therefore, the increasing the acquisition function is the same as increasing the function f , as presented in **Equation 13**:


(13) $$x^{\prime }=arg\;\underset{xєA}{\max}\;u(x│D)$$

**Figure 5:**
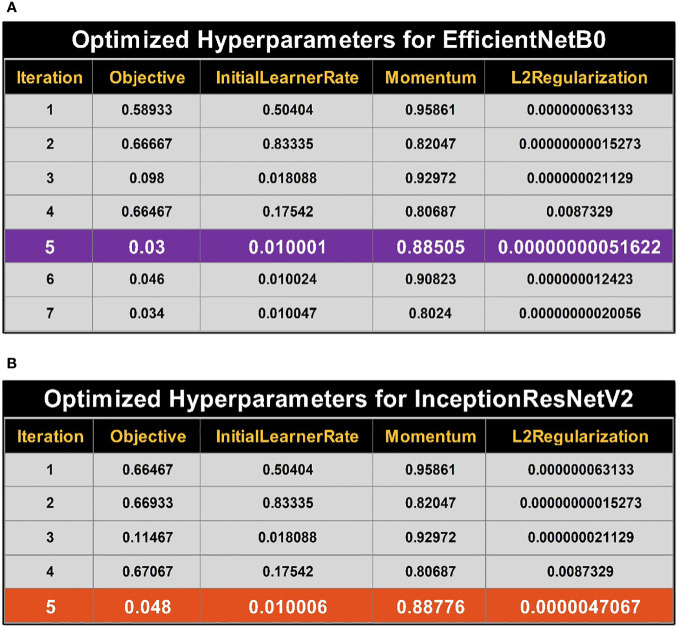
Summary of best selected hyperparameter values using BO. **(A)** Bayesian optimized (BO) hyperparameters for training of EfficientNetB0. **(B)** Bayesian optimized (BO) hyperparameters for training of InceptionResNetV2.

The acquisition function named expected improvement per second plus is employed for hyperparameter optimization. The family of acquisition functions known as “expected improvement” assesses the expected rate of improvement in the objective function while ignoring values that increase the objective. The equation for expected improvement is defined in **Equation 14**:


(14) EI(x,Q)=EQ[max(0,μQ(xbest)−f(x))]

where xbest is the location of the lowest posterior mean and μQ(xbest) is the lowest value of the posterior mean. The anticipated improvement per second used by the acquisition function during the objective function assessment is formulated in **Equation 15**:


(15) $$EIpS\left(x\right)=\frac{EIQ\left(x\right)}{\mu S\left(x\right)}$$

where μS(x) is the posterior mean of the timing Gaussian process model. Finally, the maximization process was performed and returned the best hyperparameter value. The initial learn rate (InitialLearnRate) range is 0.01–0.9, the momentum value is selected between 0.8 and 0.98, and the L2Regularization range is 1e−10 (0.0000000001) to 1e−2 (0.01) . To find the values of the hyperparameters, the search space needs to be transformed logarithmically. A logarithmic transformation is used to improve the search process order-of-magnitude balance. Results for optimizing the hyperparameters for EfficientNetB0 are provided in **Figure 5A**. While optimizing the hyperparameters, the best objective function value is achieved during iteration number 5.

Optimizing results for hyperparameters of the InceptionResNetV2 model are provided in **Figure 5B**. The best object value (i.e., optimized hyperparameters) is achieved at iteration number 5, the best and last iteration per already defined termination criteria.

### 3.5 Training and feature extraction

Both fine-tuned models have been trained on the augmented dataset, and deep features are extracted from the global average pooling layer. The sigmoid activation function has been employed in the feature extraction process and obtained a feature vector of N×1280 and N×1536 from fine-tuned EfficientNetb0 and fine-tuned InceptionResNetV2, respectively. The complex patterns are captured from the deeper layers of the above models, and higher spatial dimensions are achieved. The Global Average Pooling layer reduces the higher dimensions to a fixed-size vector; however, optimizing the features’ size for accurate classification is necessary.

### 3.6 Improved MPA optimization

In this work, we proposed an improved Quantum Theory-based Marine Predator Algorithm to select the best features. The MPA is a metaheuristics algorithm. Random walk describes the behavior of particles or objects in various physical and biological domains. These are effective methods for studying the movement of organisms such as bacteria or animals looking for food. The random character of each step in these circumstances allows for a realistic picture of how these organisms explore and navigate their surroundings. Lévy and Brown’s movements are random walks. Different velocity ratios are extracted and used in the three phases of MPA. These are strategies behind MPA (38). MPA is based on population as many other metaheuristic algorithms. The initial solution is homogeneously disseminated over the entire search space through the first sample see, **Equation 16**.


(16) $$Y_{0}=Y_{min}+rand\;\left(Y_{max}-Y_{min}\right)$$

Upper and lower bounds of variables are represented with $Y_{max}$ and $Y_{min},$ respectively, whereas uniform random vector is denoted by rand whose range is between 0 and 1 .

According to the notion of survival of the fittest, top natural predators are better foragers. As a result, the top predator, also known as elite, in the E matrix is chosen as the fittest solution. The chosen matrix is constructed, and arrays of the matrix provide a detail of searching and finding the prey based on the position of the prey. The matrix is given in **Equation 17**:


(17) $$E=\;{\left[\begin{array}{c} Y_{1,1}^{I}\;\;Y_{1,2}^{I}\;\ldots \;\;Y_{1,d}^{I}\\ Y_{2,1}^{I}\;\;Y_{2,2}^{I}\;\ldots \;\;Y_{2,d}^{I}\\ .\;\;\;\;\;\;.\;\;\;\;\;\;\;\;\;\;.\\ .\;\;\;\;\;\;.\;\;\;\;\;\;\;\;\;\;.\\ .\;\;\;\;\;\;.\;\;\;\;\;\;\;\;\;\;.\\ Y_{n,1}^{I}\;\;Y_{n,2}^{I}\;\ldots \;\;Y_{n,d}^{I} \end{array}\right]}_{n\;X\;d}$$

In the above matrix, $\vec{Y^{1}}$ is a vector representing the top predator, and it is repeated n times to create the E matrix. Dimensions are represented by d, whereas search agents are denoted by n . Predators and prey are considered search agents because predators look for its prey and the prey is looking for its food. The E matrix is updated once a better predator replaces the existing top predator.

Another matrix with the same dimensions is constructed depending on the position of the prey. Predator updates the position based on the prey’s position matrix. The matrix is named P and is given in **Equation 18**:


(18) $$P=\;{\left[\begin{array}{c} Y_{1,1}\;\;Y_{1,2}\;\ldots \;\;Y_{1,d}\\ Y_{2,1}\;\;Y_{2,2}\;\ldots \;\;Y_{2,d}\\ .\;\;\;\;\;\;.\;\;\;\;\;\;\;\;\;\;.\\ .\;\;\;\;\;\;.\;\;\;\;\;\;\;\;\;\;.\\ .\;\;\;\;\;\;.\;\;\;\;\;\;\;\;\;\;.\\ Y_{n,1}\;\;Y_{n,2}\;\ldots \;\;Y_{n,d} \end{array}\right]}_{n\;X\;d}$$

In P matrix $Y_{i,j}$ the j represents the $j^{\text{th}}$ dimension, and i represents the $i^{\text{th}}$ prey. These two matrices are the backbone for optimization.

There are three phases of MPA. These are based on the predator and prey’s life cycle and velocity criteria. These three phases are discussed separately as follows: In the first phase, the predator is considered moving faster than the prey, which is also called as the high-velocity ratio (velocity ≥ 10) phase. The ideal predator strategy is to remain still. The mathematical model for this phase is defined in **Equation 19**:


$$While\;Iteration\;<\;\frac{1}{3}\;Max\_Iteration$$

$$\vec{stepsize_{i}}=\;\vec{R_{B}}\;⊗\left(\vec{E_{i}}-\;\vec{R_{B}}\;⊗\;\vec{P_{i}}\right)\;\;i=1,\ldots n$$

(19) $$\vec{P_{i}}=\vec{P_{i}}+T.\vec{R}⊗\vec{stepsize_{i}}$$

This scenario occurs in the first third of iterations. Iteration represents the current iteration, whereas Max_Iteration represents maximum iterations. $R_{B}$ is a vector containing random values from the normal distribution exhibiting Brownian movement. Entry-wise multiplications are denoted by ⊗ . Movement of prey is simulated by the multiplication of $\vec{R_{B}}\;⊗\;\vec{P_{i}}$ . Here, T is a constant, and its value is 0.5 . R denotes a vector of uniform random numbers between 0 and 1 .

The second phase occurs in unit velocity ratio or when the prey and predator move at the same speed. It means that the predator is actively looking for prey, and the prey is actively looking for its food. This optimization stage is where the transition from exploration to exploitation occurs. The prey does exploitation, whereas exploration is the predator’s primary goal. Half of the population is designated for exploitation and the other half for exploration. If the velocity ratio ( velocity ≈ 1 ), then the prey moves in Lévy and the predator follows the Brownian motion. A mathematical model for this is given below:


$$While\;\frac{1}{3}\;Max\_Iteration\;<\;Iteration\;<\frac{2}{3}\;Max\_Iteration$$

The first half of the population can be modeled by **Equation 20**:


$$\vec{stepsize_{i}}=\;\vec{R_{L}}\;⊗\left(\vec{E_{i}}-\;\vec{R_{L}}\;⊗\;\vec{P_{i}}\right)\;\;i=1,\ldots \frac{n}{2}$$

(20) $$\vec{P_{i}}=\vec{P_{i}}+T.\vec{R}⊗\vec{stepsize_{i}}$$

In the above equation, $\vec{R_{L}}$ represents the Lévy movement of the first half of the population. The multiplication of $\vec{R_{L}}\;⊗\;\vec{P_{i}}$ describes the Lévy movement of the prey, and adding the step size of the prey position determines its movement. The second half of the population can be modeled in the given below **Equation 21**:


$$\vec{stepsize_{i}}=\;\vec{R_{B}}\;⊗\left(\vec{R_{B}}⊗\;\vec{E_{i}}-\;\vec{P_{i}}\right)\;\;i=\frac{n}{2},..,n$$

(21) $$\vec{P_{i}}=\vec{E_{i}}+T.\vec{CF}⊗\vec{stepsize_{i}}$$

where $CF={\left(1-\frac{Iteration}{Max\_Iteration}\right)}^{\left(2\frac{Iteration}{Max\_Iteration}\right)}$ is regarded as an adaptive parameter to regulate the predator’s movement’s step size. The multiplication of $\vec{R_{B}}⊗\;\vec{E_{i}}$ determines the step size in the Brownian movement of the predator, whereas the prey modifies its position in relation with the predator’s movement. The third phase starts with a low velocity ratio or when a predator has a faster pace than the prey. It is the last phase of optimization. High exploitation capability is demonstrated in this phase. In such a low-velocity ratio of velocity= 0.1, the predator adopts the Lévy strategy. The mathematical model is provided in **Equation 22**:


$$While\;Iteration>\;\frac{2}{3}\;Max\_Iteration$$

$$\vec{stepsize_{i}}=\;\vec{R_{L}}\;⊗\left(\vec{R_{L}}⊗\;\vec{E_{i}}-\;\vec{P_{i}}\right)\;\;i=1,..,n$$

(22) $$\vec{P_{i}}=\vec{E_{i}}+T.\vec{CF}⊗\vec{stepsize_{i}}$$

In the Lévy method, multiplying $\vec{R_{L}}⊗\;\vec{E_{i}}$ simulates the predator’s movement, whereas adding the step size to the Elite position assists in updating the position of the prey. Fish aggregating devices (FADs), considered local optima in their search space, are where sharks spend most of their time (i.e., more than 80% of the time). They make longer jumps in diverse directions during the remaining 20% of their time, probably to locate different prey distributions. To ensure a more dynamic search during the simulation, these lengthier hops help prevent them from being stuck in local optima. The FAD effect’s mathematical elaboration can be represented as the following **Equation 23**:


(23) $$\vec{P_{i}}=\;\{\begin{array}{c} \vec{P_{i}}+CF\left[\vec{Y_{min}}+\;\vec{R}\;⊗\;\left(\vec{Y_{max}}-\;\vec{Y_{min}}\right)\right]\;⊗\;\vec{U}\;\;\;\;if\;r\;\leq FADs\\ \vec{P_{i}}+\;\left[FADs\;\left(1-r\right)+r\right]\;\left(\vec{P_{r1}}-\;\vec{P_{r2}}\right)\;\;\;\;\;\;\;\;\;\;\;\;if\;r\;\geq FADs \end{array}$$

The likelihood that FADs may affect the optimization process is represented by the probability, given as 0.2 . A binary vector $\vec{U}$ is made up of zeros and ones. It is created by a random vector with values between [0,1], with a zero set for values below 0.2 and one for values above 0.2. Additionally, r stands for a random number uniformly distributed between [0,1]. $\vec{Y_{min}}$ and $\vec{Y_{max}}$ denote lower and upper bounds of dimensions. P matrix’s random indexes are denoted by subscripts $r_{1}$ and $r_{2}$ .

**Novelty in this method**

The problem of the MPA algorithm is finding an optimal global position; therefore, we added a concept of Quantum Theory that improves populations’ motion behavior. The initial population in the modified version is defined as follows:


$$Z_{i}\left(k+1\right)=Z_{min}+r\times \left[Z_{max}-Z_{min}\right]$$

where $Z_{i}$ denotes the ith iteration value, r is a random value between (0,1), k is a current iteration, and $Z_{max}$ and $Z_{min}$ denote the upper and lower limits, respectively. The fitness value is computed to find the best solution in the next step. The following **Equations 24**–**27** is utilized for this purpose:


(24) $${\vec{L}}_{Q}=Z_{i,j}\left(k+1\right)=\{\begin{array}{c} C_{i}-b*\left(J_{best}-Z_{i,j}\left(k\right)\right)\times \ln\left(\frac{1}{u}\right),\;\;\;if\;T\geq Entropy\\ C_{i}+b*\left(J_{best}-Z_{i,j}\left(k\right)\right)\times \ln\left(\frac{1}{u}\right),\;\;\;if\;T\geq Entropy \end{array}$$

(25) $$Q\left(Z_{i,k+1}^{j}\right)=\frac{1}{Length_{i,k}^{j}}exp\left(-\frac{2\left|z_{i,k+1}^{j}-s_{i,k}^{j}\right|}{Length_{i,k}^{j}}\right)$$

(26) $$C_{i}=\theta \times cBest_{i}+\left(1-\theta \right)\times ɡBest$$

(27) $$J_{best}=\frac{1}{N}\sum_{i=1}^{N}cBest_{i}$$

The notation $cBest_{i}$ denotes the best position in the ith iteration for the predator, and gBest is the best position for all predators at each iteration. The average best predator is denoted by $J_{best}$ , and θ is the distribution of a chaotic number on (0,1). The b denotes the contraction expression phase, and it is used to control the convergence rate. Mathematically, b is defined by **Equation 28**:


(28) $$b=b_{max}-\left[\left\{\frac{b_{max}-b_{min}}{Iterations_{max}}\right\}\times Iterations\right]$$

Hence, the final equation is formulated as the following **Equation 29**:


(29) $$\vec{Step}={\vec{C}}_{Q}⨂\left({\vec{Elite}}_{i}-{\vec{C}}_{Q}⊗{\vec{Prey}}_{i}\right),\;i=1,2,\ldots \frac{n}{2}$$

Every solution in the current iteration is compared with its equivalent in the prior iteration for fitness. If the current solution is found to be a superior match, the previous one is superseded. This iterative procedure improves solution quality with time and imitates the behavior of predators that return to locations with abundant prey after successful foraging attempts. After completion of optimization, a feature vector of dimension N×380  and a feature vector of N×313 , respectively, are obtained.

### 3.7 Feature fusion and classification

The selected features are finally fused and later classified using machine learning classifiers. The fusion process improves an object’s information that directly relates to better accuracy. In this work, a simple serial-based fusion has been chosen to combine the selected feature vectors in a single vector.

Using the following equation, we can determine the dimension of the serial-based fusion vector if we have two feature vectors, f1 and f2 , with dimensions of N×380 and N×313 , respectively, where N denotes the total number of observations as defined by **Equation 30**.


(30) $$R=\;{\left(\begin{array}{c} f1\\ f2 \end{array}\right)}_{N\times 380\;+N\times \;313}$$

The resultant feature vector is obtained of dimension N×693 . The fused feature vector is finally classified using traditional machine learning classifiers named as Cubic SVM and Weighted KNN and neural network-based classifiers such as narrow, wide, tri-layered, bi-layered, and medium. The hyperparameters used to train these classifiers are provided in **Table 1** as follows:

**Table 1:** Classifiers and training hyperparameters of each classifier.

Classifier	Training hyperparameters	Classifier	Training hyperparameters
**Cubic SVM**	Kernel function: Cubic Kernel scale: Automatic Box constraint level: 1 Multiclass method: one-vs-one Standardize data: true	**Weighted KNN**	Number of neighbors: 10 Distance metric: Euclidean Distance weight: squared inverse Standardize data: true
**Wide neural network**	Number of fully connected layers: 1 First layer size: 100 Activation: ReLU Iteration limit: 1000 Regularization strength (Lambda): 0 Standardize data: yes	**Medium neural network**	Number of fully connected layers: 1 First layer size: 25 Activation: ReLU Iteration limit: 1000 Regularization strength (Lambda): 0 Standardize data: yes
**Bilayered neural network**	Number of fully connected layers: 2 First layer size: 10 Second layer size: 10 Activation: ReLU Iteration limit: 1000 Regularization strength (Lambda): 0 Standardize data: yes	**Trilayered neural network**	Number of fully connected layers: 3 First layer size: 10 Second layer size: 10 Third layer size: 10 Activation: ReLU Iteration limit: 1000 Regularization strength (Lambda): 0 Standardize data: yes
**Narrow neural network**	Number of fully connected layers: 1 First layer size: 10 Activation: ReLU Iteration limit: 1000 Regularization strength (Lambda): 0 Standardize data: yes		

### 3.8 Dataset and performance evaluation

The augmented Figshare dataset is used for our experiments and is contributed by (11). The dataset is publicly available for research purposes. A model or algorithm’s ability to predict outcomes based on the available data is measured using performance metrics in machine learning. The calculated measures contain each classifier’s sensitivity rate, false negative rate (FNR), precision rate, and area under the curve (AUC). Time and accuracy measures are also used to interpret the performance of each classifier. **Table 2** provides more details on these performance metrics.

**Table 2:** Performance measures used to validate the proposed methodology.

Name	Accuracy (%)	Time	Sensitivity rate (%)	False negative rate (%)	Precision rate (%)	Area under the curve
**Performance measure**	$\frac{TP+TN}{TP+TN+FP+FN}$	*Seconds*	$\frac{TP}{TP+FP}$	$\frac{FN}{TP+FP}$	$\frac{TP}{TP+FN}$	$\int_{a}^{b}f\left(x\right)\text{d}x$
**Description**	It is a characteristic or condition of being precise or accurate.	Precise time to finish a task	Measures how successfully a test finds true positives.	Probability of failure to detect a true positive.	Degree of false positives in the result	x-axis integral over a particular time

TP is for true positive, TN is for true negative, FP is for false positive, and FN is for false negative.

The reason to choose each measure provided in **Table 1** is given below:

• Accuracy is the ratio of accurately predicted occurrences to total instances. This gives a general idea of how well a model is predicting in every class. Accuracy by itself, though, could not be enough if the classes are unbalanced. In our proposed technique, classes are balanced. Balance among classes is achieved by data augmentation process.
• Time required to finish a specific task is given in seconds.
• Sensitivity quantifies the percentage of actual positive instances that the model accurately predicted. In order to reduce false negatives, it is very crucial. For example, in the medical domain, high sensitivity indicates that the model is effective in identifying positive cases.
• False negative rate refers to the percentage of true positive cases that were mistakenly forecast as negative. It stands for the probability of overlooking favorable examples. When the cost of missing positive occurrences is large, it is essential to reduce false negative rate.
• Precision gauges how well the model predicts positive occurrences. If you wish to reduce false positives, accuracy is crucial. For instance, high precision in medical diagnosis indicates that the model is likely to be accurate when it predicts a positive case.
• The area under the receiver operating characteristic (ROC) curve is known as the area under the curve (AUC). The trade-off between true positive rate (sensitivity) and false positive rate is represented graphically by the ROC curve. AUC offers a single scalar value that sums up the model’s overall performance. Perfect categorization is indicated by an AUC of 1.0; random chance is suggested by an AUC of 0.5.

## 4 Results and discussion

### 4.1 Experimental setup

In this section, detailed experimental setup is discussed. The data augmentation is performed using a sparse auto-encoder. A single hidden layer with 300 neurons is selected while the training parameters like a maximum epochs are 2,000, L2WeightRegularization is set equal to 0.001, SparsityRegularization is equal to 4, and finally, SparsityProportion is set to 0.15. Hyperparameter optimization is performed to optimize the parameters of fine-tuned deep models such as EfficientNetB0 and InceptionResNetV2. The original dataset is split into a ratio of 50:50 in training and test proportions. After that, the training and testing images are separately augmented and trained models. The gradient vectors are accelerated via stochastic gradient descent (SGDM) for quicker convergence at the convolutional layers. During the algorithm learning phase for both models, a mini-batch size 128 is chosen. Additionally, the experiments are carried out using MATLAB R2023a on a machine equipped with 128 GB of RAM and CPU Intel(R) Core(TM) i7-6700 @ 3.40 GHz and 12 GB RTX3000.

### 4.2 Proposed framework results (fine-tuned models)

In this section, results of the first step of the proposed framework are presented. The hyperparameter optimization using the Bayesian method is performed separately for EfficientNetB0 and InceptionResNetV2 models and numerical results are computed.

#### 4.2.1 Fine-tuned Bayesian optimization-based EfficientNetB0

**Figure 6:**
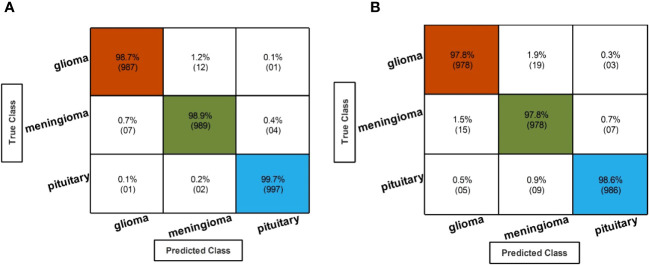
Confusion matrix of EffficientNetB0 and InceptionResNetV2 hyperparameter optimization using BO. **(A)** Confusion matrix of fine-tuned EffficientNetB0 hyperparameter optimization using BO. **(B)** Confusion matrix of fine-tuned InceptionResNetV2 hyperparameter optimization using BO.

**Table 3** describes the classification performance of fine-tuned EfficientNetB0 deep architecture with an accuracy value of 99.10%, achieved by the Cubic SVM classifier. The wide neural network obtained the second best accuracy of 98.90%. The rest of the classifiers obtained accuracies of 98.80%, 98.70%, 98.60%, and 98.50%. The sensitivity and precision rates of each classifier are also noted, and the maximum value of Cubic SVM is 99.10%. In addition, the performance of Cubic SVM can be confirmed by a confusion matrix, given in **Figure 6A**. The diagonal numbers in the figure represent the number of true observations and the true positive rate for glioma, meningioma, and pituitary classes. The computational time of each classifier is also noted during the classification process, and it is observed that the minimum noted time is 12.512 (seconds) for Medium Neural Network.

**Table 3:** Classification results using the BO-based EfficientNetB0 model.

Classifier	Accuracy (%)	Time (s)	Sensitivity rate (%)	False negative rate (%)	Precision rate (%)	Area under curve (%)
**Cubic SVM**	**99.10**	20.227	**99.10**	**0.90**	**99.10**	**1.00**
**Wide neural network**	98.90	14.505	98.93	1.07	98.93	1.00
**Medium neural network**	98.80	**12.512**	98.76	1.24	98.76	1.00
**Bilayered neural network**	98.70	15.886	98.66	1.34	98.66	0.99
**Weighted KNN**	98.60	26.907	98.63	1.37	98.63	1.00
**Narrow neural network**	98.60	15.377	98.60	1.40	98.60	0.99
**Trilayered neural network**	98.50	16.059	98.53	1.47	98.53	1.00

Bold denotes the best values.

#### 4.2.2 Fine-tuned Bayesian optimization-based InceptionResNetV2

In the second step, the classification results are computed using fine-tuned InceptionResNetV2 with the initialization of BO-based hyperparameters. The results of this step are given in **Table 4**, which shows the maximum accuracy of 98.10 for the Cubic SVM classifier. The minimum computational time of this step is 20.543 (second) for the Narrow Neural Network classifier. In addition, the performance of the Cubic SVM classifier can be confirmed by a confusion matrix, illustrated in **Figure 6B**. Compared with the performance of this step with step 1, it is observed that the accuracy of this step is degraded by approximately 1%. Moreover, the increase in time shows the drawbacks of this step. In order to reduce the drawbacks of this step, a feature selection method is employed, which selects only important features for classification.

**Table 4:** Classification results of using BO-based InceptionResNetV2.

Classifier	Accuracy (%)	Time (s)	Sensitivity rate (%)	False negative rate (%)	Precision rate (%)	Area under curve (%)
**Cubic SVM**	**98.10**	21.966	**98.06**	**1.94**	**98.06**	**1.00**
**Narrow neural network**	97.90	**20.543**	97.93	2.07	97.93	0.99
**Wide neural network**	97.90	49.824	97.93	2.07	97.93	1.00
**Bilayered neural network**	97.90	36.736	97.93	2.07	97.93	0.99
**Trilayered neural network**	97.90	36.711	97.86	2.14	97.86	0.99
**Medium neural network**	97.80	29.453	97.80	2.20	97.80	1.00
**Weighted KNN**	97.10	34.365	97.13	2.87	97.13	1.00

Bold denotes the best values.

### 4.3 Feature selection using proposed QTbMPA feature selection

The third and fourth steps correspond to the best feature selection.

#### 4.3.1 QTbMPA feature selection on fine-tuned EfficientNetB0

**Figure 7:**
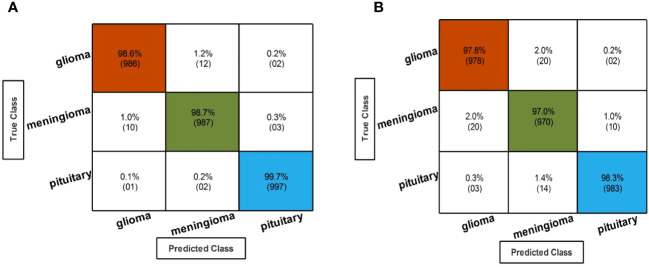
Confusion matrix of the QTbMPA selection technique for EffficientNetB0 and InceptionResNetV2. **(A)** Confusion matrix of QTbMPA based best selected EfficentNetB0 deep features. **(B)** Confusion matrix of QTbMPA based best selected InceptionResNetV2 deep features.

In the third step, the proposed feature selection method is applied to deep extracted features; in return, the best optimal features are obtained. The results of the feature selection method on fine-tuned EfficientNetB0 are presented in **Table 5**. In this table, the maximum accuracy of 99.00% by the Cubic SVM classifier is shown. The sensitivity and precision rate of this classifier are also 99% that the confusion matrix in **Figure 7A** can confirm. Wide neural network obtained the second best accuracy of 98.80%. Each classifier’s computational time is noted, and its minimum reported time is 3.8078 (sec). In step 1, the minimum time was 12.52 (s), which is now reduced by almost 300%. Moreover, the accuracy of this step is consistent, which can be a strength of the proposed feature selection method.

**Table 5:** Proposed classification results after employing the QTbMPA selection method on features returned from the Bayesian-based EfficientNetB0 model.

Classifier	Accuracy (%)	Time (s)	Sensitivity rate (%)	False negative rate (%)	Precision rate (%)	Area under curve (%)
**Cubic SVM**	**99.00**	5.4668	**99.00**	**1.00**	**99.00**	**1.00**
**Wide neural network**	98.80	4.5414	98.83	1.17	98.83	1.00
**Medium neural network**	98.60	3.8831	98.63	1.37	98.63	1.00
**Weighted KNN**	98.60	7.4494	98.56	1.44	98.56	1.00
**Trilayered neural network**	98.50	4.9574	98.53	1.47	98.53	0.99
**Narrow neural network**	98.50	3.9573	98.50	1.5	98.50	1.00
**Bilayered neural network**	98.50	**3.8078**	98.50	1.5	98.50	0.99

Bold denotes the best values.

#### 4.3.2 QTbMPA feature selection on fine-tuned InceptionResNetV2

In the fourth step, features of the fine-tuned InceptionResNetV2 model are selected using the proposed QTbMPA method and classification is performed. **Table 6** describes the results of this step, showing an maximum accuracy of 97.70% by Cubic SVM. The sensitivity and precision rate of this classifier is also 97.70%. The confusion matrix in **Figure 7B** can further confirm these values. The computational time of each classifier is also given in this table, and the minimum reported time is 6.1486 (s) for Cubic SVM. Compared with the computational time of this step with the second step, the time is reduced almost 300%.

**Table 6:** Proposed classification results after employing the QTbMPA selection method on features returned from the Bayesian-based InceptionResNetV2 model.

Classifier	Accuracy (%)	Time (s)	Sensitivity rate (%)	False negative rate (%)	Precision rate (%)	Area under curve (%)
**Cubic SVM**	**97.70**	**6.1486**	**97.70**	**2.30**	**97.70**	**1.00**
**Narrow neural network**	97.60	7.8251	97.60	2.40	97.60	0.99
**Medium neural network**	97.60	7.3787	97.60	2.40	97.60	0.99
**Bilayered neural network**	97.50	7.7592	97.50	2.50	97.5	0.98
**Wide neural network**	97.30	11.376	97.20	2.80	97.20	0.99
**Trilayered neural network**	97.30	8.9962	97.26	2.74	97.26	0.99
**Weighted KNN**	97.20	6.8459	97.16	2.84	97.16	0.99

Bold denotes the best values.

#### 4.3.3 Fusion of best selected features

**Figure 8:**
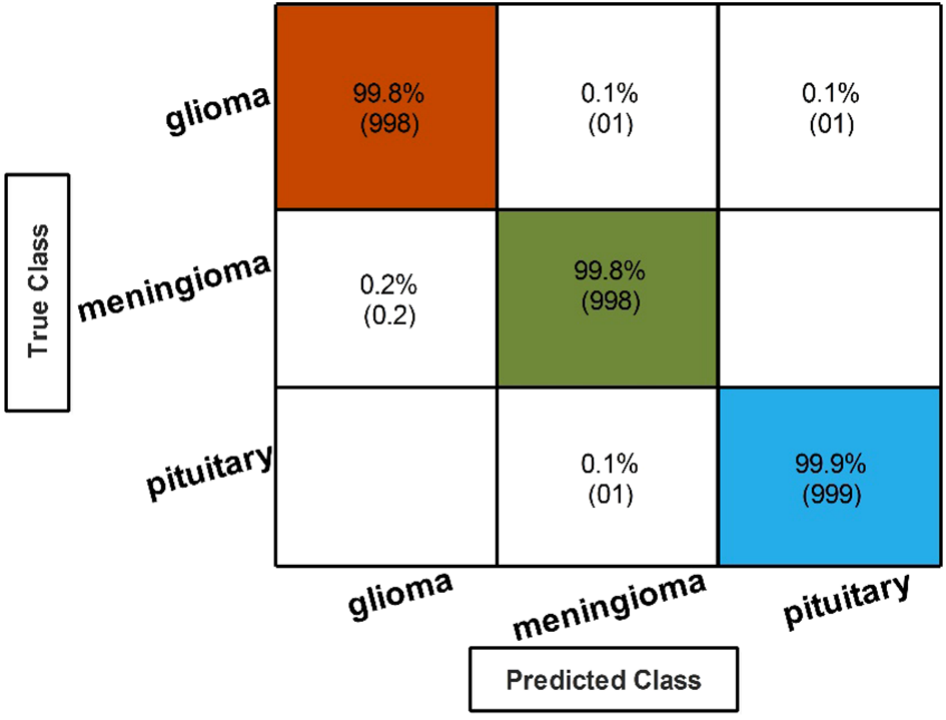
Classification results after the fusion of selected feature features.

Finally, the best-selected features of both models, in the third and fourth steps, are fused using a serial approach. The cubic SVM classifier obtained the maximum accuracy of 99.80% and the sensitivity and precision rates of 99.83% *(can be seen in* ***Table 7*** *)*. The confusion matrix in **Figure 8** can further confirm these values. A minor increase in computational time is observed after the fusion process; however, the accuracy is significantly improved for all classifiers. In comparison, with all previous steps, noted accuracy has significantly improved and is the highest among all early noted accuracies. Moreover, **Table 8** shows a detailed comparison of the proposed method with state-of-the-art techniques and shows significant improvement.

**Table 7:** Classification results after fusing of the best selected features of both models.

Classifier	Accuracy (%)	Time (s)	Sensitivity rate (%)	False negative rate (%)	Precision rate (%)	Area under curve (%)
**Cubic SVM**	**99.80**	11.198	**99.83**	**0.17**	**99.83**	**1.00**
**Weighted KNN**	99.80	13.699	99.80	0.20	99.80	1.00
**Wide neural network**	99.80	6.776	99.76	0.24	99.76	1.00
**Medium neural network**	99.70	5.6234	99.70	0.30	99.70	1.00
**Bilayered neural network**	99.70	**5.0002**	99.66	0.34	99.66	1.00
**Trilayered neural network**	99.70	5.6399	99.66	0.34	99.66	1.00
**Narrow neural network**	99.60	6.2125	99.60	0.40	99.60	1.00

Bold denotes the best values.

**Table 8:** Summary of recent state-of-the-art (SOTA) techniques for brain tumor classification using Figshare dataset.

Serial no.	Reference	Year	Dataset	Accuracy (%)
1	Alanazi et al. (19)	2022	Figshare	95.75
2	Raza et al. (20)	2022	Figshare	99.67
3	Tummala et al. (21)	2022	Figshare	98.70
4	Polat et al. (22)	2022	Figshare	99.18
5	Shaik et al. (23)	2022	Figshare	96.51
6	Haq et al. (24)	2022	Figshare	98.00
7	Rahman et al. (25)	2023	Figshare	97.60
8	Talukder et al. (47)	2023	Figshare	99.68
9	Aloraini et al. (26)	2023	Figshare	99.10
10	Athisayamani et al. (27)	2023	Figshare	98.85
11	Mishra et al. (28)	2023	Figshare	98.97
12	Agrawal et al. (48)	2023	Figshare	96.40
13	Malla et al. (49)	2023	Figshare	98.93
14	Asif et al. (50)	2023	Figshare	98.69
15	Cinar et al. (29)	2023	Figshare	98.32
16	Deepak et al. (30)	2023	Figshare	95.40
17	Zulfiqar et al. (31)	2023	Figshare	98.86
18	Shyamala et al. (51)	2023	Figshare	94.70
19	Yapici et al. (52)	2023	Figshare	99.47
20	Sahoo et al. (53).	2023	Figshare	97.00
	**Proposed**		Figshare	**99.80**

Bold denotes the best values.

### 4.4 Discussion

A t-test is a statistical technique used to compare the mean values of two groups. It is frequently used in hypothesis testing to see whether a particular process or treatment has a noticeable effect on the target population or whether there is a significant difference between the two groups. In order to test the significant difference between the classifiers, t-test is applied.

In the proposed technique, t-test is conducted to check any considerable gap between accuracies at different stages of our proposed model. The gap is resulted when we have unbalanced classes of dataset (54). The augmented step balanced the classes of dataset; however, to validate our augmentation step, t-test is applied on all phases of the proposed technique. The test starts by setting a null hypothesis as below:


$$\begin{array}{c} H_{0}\;=\;The\;accuracy\;of\;the\;chosen\;classifiers\;differs\;significantly\;\\ over\;the\;phases\;of\;proposed\;technique. \end{array}$$

Additionally, two best-performing classifiers at all phases of the proposed technique are chosen. The accuracy achieved by respective classifier at each phase is selected to conduct experiments.

A detailed overview of test is given below:

**Table:** 

Phases →	BO based EfficientNetB0	BO based InceptionResNetV2	MPA Optimization for EfficientNetB0	MPA Optimization for InceptionResNetV2	Feature fusion
Classifiers ↓					
**Cubic SVM**	99.10	98.10	99.00	99.70	99.80
**Weighted KNN**	98.60	97.10	98.60	97.20	99.80

The mean of the differences for all experiments are calculated using the following Equations 31–34:


(31) Difference (J)= |Accuracy(k)−Accuracy(l)|

(32) $$Mean\;\left(\mu \right)=\;\frac{1}{N}\sum_{k=1}^{I}\left|J_{k}\right|$$

where I is the number of experiments and the noted mean value after this step is 0.48 . After calculating the Mean (μ) , the standard deviation (σ) is calculated by using the following equation:


(33) $$standard\;deviation\;\left(\sigma \right)=\;\sqrt{\frac{{\left(\sum_{k=1}^{I}\left(J_{k}\right)-\mu \right)}^{2}}{I-1}}$$

The resultant standard deviation value is 0.357, later used in the T Selection formula.


(34) $$\;T\;Selection=\frac{\sqrt{I\;}\times \;\mu \;}{\sigma }$$

The value 3.012 was obtained after calculation using the above formula. The obtained value will be considered as a decisive point to conduct the Student's T−Test . Moreover, the degree of freedom (df) is calculated using the formula: df=n−1 ; the resultant value is four and selected p value = 0.05 (55). After looking at the corresponding output value in the t-test chart, the value is (−2.776, +2.776) . The decisive T Selection value is 3.012 ; based on the given below formulation in equation 35, it is established that $H_{0}$ is rejected, and there is no noteworthy difference between the atp10ccuracy of the selected classifiers.


(35) If (T Selection >= −2.776 and <= + 2.776)

Hypothesis test establishes that throughout the phases of the proposed technique, there is a consistency in accuracy of each phase; it means that the class imbalance problem is accurately addressed. Inconsistent accuracies are the result of imbalance classes of dataset, which lead to loss of accuracies. The proposed data augmentation step helps to properly address class imbalance problem.

Heat map-based analysis: Heat map-based techniques are employed to express the decisive features of classification for each class. Grad-CAM, LIME, and Occlusion Sensitivity are three methods commonly used to represent decision features for classification of an image. Grad-CAM uses gradients to determine the classification score about the final convolutional feature map. It draws attention to that part of input image that has the biggest influence on this score. The method uses a global average pooling layer to extract features. Equation 36 serves as the basis for this procedure, which is illustrated below:


(36) $$b_{ɡ}^{c}=\;\frac{1}{N}\;\sum_{i}\sum_{j}\frac{\partial_{y}^{c}}{\partial B_{i,j}^{ɡ}}$$

where $b_{g}^{c}$ represents class scores of ɡ features from the Global Average Pooling layer, N represents total pixels in a feature map, c depicts the class score, and y is the considered output. The whole expression $\partial B_{i,j}^{ɡ}$ represents the convolution map. In the expression, i and j represent two dimensions and B represents gradients. Features with negative weight can be possible using the above equation; therefore, the Relu activation function is used to convert the negative weights to positive and is represented using the given below Equation 37:


(37) $$\text{M}=\text{Relu}\left(\sum^{b_{g}^{c}}\;.\;B^{g}\right)\;$$

**Figure 9:**
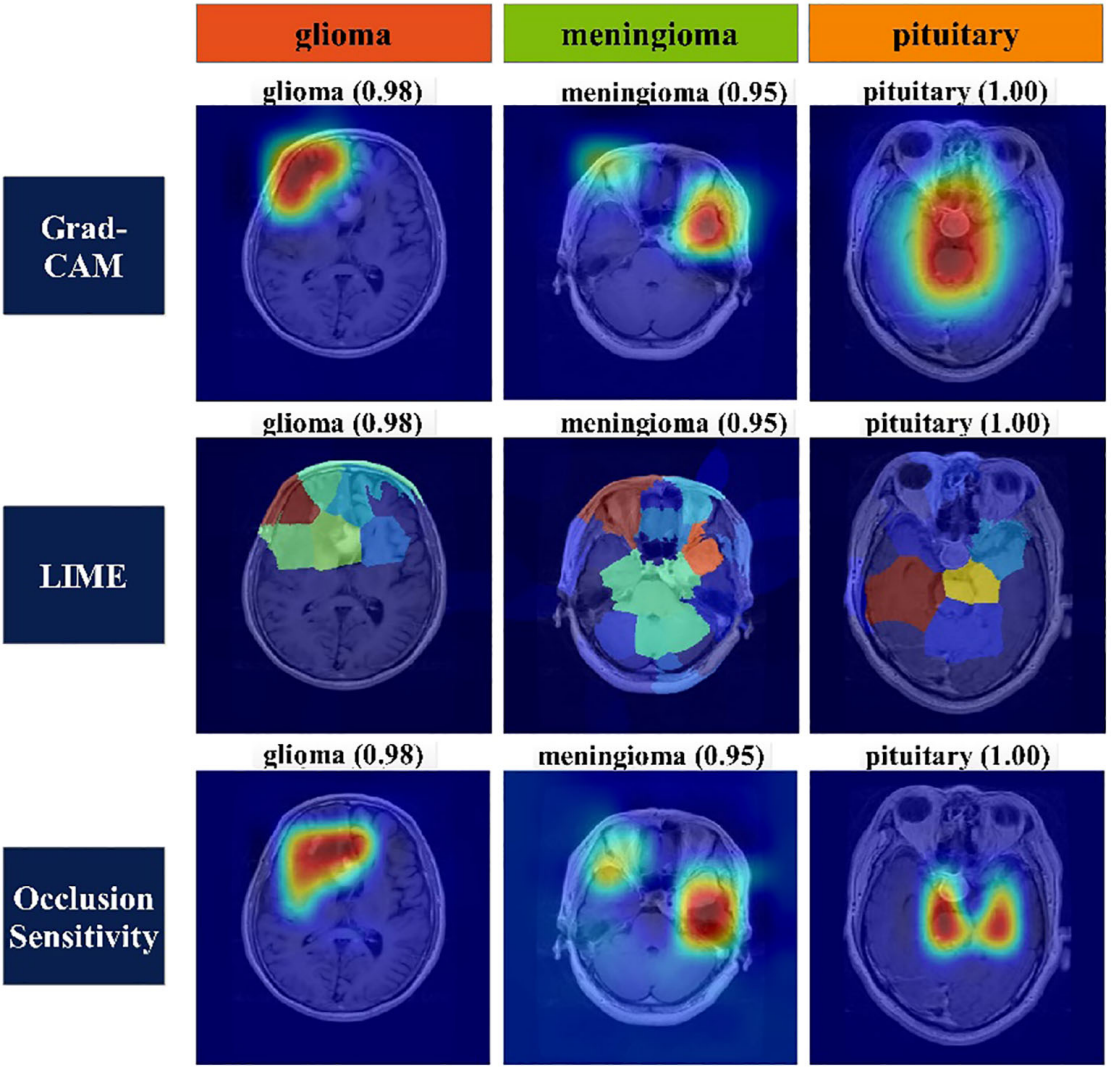
Heat map of classes using Grad-CAM, LIME, and occlusion sensitivity.

Mathematical details of LIME and Occlusion Sensitivity can be seen from (56) and (57), respectively. **Figure 9** represents the visualization of important features of each class using the explained methods of the heat map.

## 5 Conclusion

This article presents a novel deep learning framework with an efficient QTbMPA feature selection technique for the classification of brain tumor types such as meningioma, glioma, and pituitary from MRI images. Instead of manual data augmentation, a sparse autoencoder architecture was proposed and generated new images based on the training set. Two lightweight deep learning architectures were modified and trained with the help of BO hyperparameter initialization. The deeper layer (global average pool) was employed for feature extraction and performed classification. The classification process shows that there exist few irrelevant features, which are impacted on the classification computational time. Therefore, we proposed an efficient QTbMPA feature selection algorithm that almost 300% reduced the computational time and maintained the classification accuracy. The selected features were finally fused and classified using ML and neural network classifiers. On the augmented dataset, the proposed framework obtained an improved accuracy of 99.80% than the SOTA technique.

The goal of the proposed research is to create a deep learning (DL) model for brain tumor classification, utilizing DL’s capabilities to classify various forms of brain tumors more accurately. This finding could have a significant clinical impact in neuro-oncology and have a wide variety of potential applications. The proposed research can assist doctors and radiologists in making accurate diagnoses when using medical imaging data, such as MRI scans, to identify brain tumors. It offers dependable and consistent tumor categorization results, lowering the misdiagnosis risk and enabling early brain tumor discovery. Furthermore, the accurate classification of brain tumors might help in developing customized treatment plans for patients. The model assists physicians in developing customized treatment regimens that lead to more accurate and successful treatment outcomes by aiding in the identification of the exact type of tumor.

### 5.1 Limitations and future work

Although we obtained the maximum accuracy, there are few limitations that make the proposed architecture more consistent. The limitations of this work are selection of pretrained models and best feature selection. The pretrained models have been selected based on the Top-5 accuracy on ImageNet dataset and total number of parameters. In addition, the selection process reduces the overfitting, but still there are few irrelevant features selected for the classification. The proposed architecture has been evaluated on brain tumor MRIs of the Figshare dataset; however, in future, it will be tested on BRATS datasets. Moreover, a new self-attention and vision transformer model will be proposed for the improved accuracy and efficiency.

## Data availability statement

Publicly available datasets were analyzed in this study. This data can be found here: https://www.kaggle.com/datasets/masoudnickparvar/brain-tumor-mri-dataset.

## Author contributions

MU: Data curation, Methodology, Conceptualization, Investigation, Project administration, Software, Writing – original draft. MK: Conceptualization, Investigation, Methodology, Software, Supervision, Writing – original draft. AM: Conceptualization, Formal analysis, Methodology, Software, Writing – original draft. OM: Data curation, Formal analysis, Project administration, Visualization, Writing – review & editing. OS: Conceptualization, Data curation, Formal analysis, Funding acquisition, Project administration, Visualization, Writing – review & editing. NA: Project administration, Resources, Software, Supervision, Validation, Visualization, Writing – review & editing.
